# The Effect of Health Change on Long-Term Settlement Intentions of International Immigrants in New Destination Countries: Evidence from Yiwu City in China

**DOI:** 10.3390/ijerph19137574

**Published:** 2022-06-21

**Authors:** Tao Xu

**Affiliations:** College of Law and Political Science, Zhejiang Normal University, Jinhua 321004, China; xutao@zjnu.edu.cn

**Keywords:** willingness to settle down, changes in health status, social insurance, length of stay

## Abstract

**Objective:** Previous studies on settlement intentions have mainly focused on the explanations of social and economic rationality, culture, and institution, but insufficient attention had been paid to the relationship between health and settlement intentions. This study explored the relationship between changes in the health status of immigrants and their settlement intentions. **Method:** A cross-sectional survey was conducted both in 2018 and 2019. Foreigners who visited the Yiwu Municipal Exit–Entry Administration Office to extend their visas were invited to participate in the study. Quantitative data, such as the participants’ sociodemographic characteristics, job status, employment, immigration experience, key factors associated with the intention to settle down, medical insurance coverage, and changes in health status, were collected by questionnaire. **Results:** A change in health status significantly affected the intention to settle down: the more healthy that people became, the more likely they intended to settle down (β = 0.233; *p* < 0.001), and, simultaneously, changes in health status were also moderated by the length of stay in China (β = 0.320; *p* < 0.001) and medical insurance (β = 0.134; *p* < 0.001), which affected people’s willingness to settle down. **Conclusions:** Changes in health status, and not health status itself, affected immigrants’ willingness to settle down. In addition, social insurance, and the length of stay in the place receiving immigrants, affected immigrants’ willingness to settle down not only directly, but also indirectly by moderating the impact of the change in health status on the willingness to settle down.

## 1. Introduction

Settlement intention refers to immigrants’ willingness to reside permanently in the society to which they move, and a great deal of research has considered the factors that influence the settlement intentions of immigrants [1,2,3,4,5,6]. The research objects include not only migrants from rural areas to cities in the process of urbanization, but also international immigrants in the process of globalization [2,7,8]. How to design and formulate relevant policies to promote the settlement and integration of immigrants is a common problem that is faced by societies in places that receive immigrants. Therefore, the issue is also a common focus of researchers. Strictly speaking, investigating the actual settlement behavior of immigrants must rely on large-scale and longitudinal survey data. However, such data are very difficult to obtain, and especially in developing countries. Thus, an alternative approach is to understand the motivations of potential settlers and returnees by analyzing the settlement intentions of immigrants. A large number of studies have examined the determinants of immigrants’ permanent settlement [9,10,11,12].

Studies on immigrants’ willingness to settle down mainly focus on how variables, such as individuals’ socioeconomic characteristics, social integration status, and the social institution setting in the place receiving immigrants, influence their migration and residence decisions [5,6,11,13,14,15]. Few studies have examined the relationship between the health of immigrants and their settlement intentions. Although these studies confirm that there is a close correlation between the health status of immigrants and their willingness to settle down [16,17,18,19,20,21,22], they often consider health status as a constant. In fact, health status is constantly changing, and the relationship between health-status change, settlement intentions, and remigration needs to be further studied. This study uses empirical data to reveal the relationship between health-status change and residence intention.

### 1.1. Socioeconomic, Cultural, and Institutional Perspectives and Long-Term Settlement Intention

A review of the existing academic analyses on the factors that influence the settlement intentions of immigrants indicates that there are three types of perspectives: economic rationality, and cultural and institutional explanations.

From the perspective of economic rationality, the most important factor in the decisions of immigrants to settle in a certain country (region) comes from the difference between the economic returns of the inflowing country and the exporting country in terms of human capital, according to the theory of neoclassical economics [23,24]. If the economic returns of the inflowing country are better than the exporting country, for rational considerations, they will move from the exporting country to the inflowing country in order to obtain better returns [7,11]. After that, the economic integration status in the immigration country will directly affect whether immigrants make long-term settlement decisions. The more successful they are as individuals in the immigrant-receiving country, the more likely they are to settle down in the country [11]. Once the economic benefits of the immigrant-receiving place are reduced, or even lower than that of the country of origin, those who have migrated for economic reasons will migrate back to their own country again. Usually, those who have received a good education and those with stronger input language skills have more human capital and are more likely to obtain better jobs and better returns in the immigrant-receiving place. Therefore, they usually fare better than people with low education and poor language skills and are more inclined to settle down. Generally, there is a positive correlation between education level and settlement intention. The higher an individual’s education level is, the more likely he/she is to settle down in the society to which he/she immigrated. The logic behind this is that the more educated people are, the easier it is for them to find a good job in the labor market of the society, and thus, they can improve their social living conditions. In addition, their values are less fixed, they learn languages and cultural customs faster, and they can more easily integrate into the society of the immigrant-receiving place [15,25,26,27].

From the cultural perspective, the amount and quality of the social interaction in the immigrant-receiving place can significantly affect future settlement intentions [28]. In the early stage of immigration, having many relatives and friends in the society of the immigrant-receiving place helps immigrants to become established there. People from the same ethnic group and region can provide not only material help, such as a place to live, sustenance, and basic living conditions, but also emotional support to help immigrants through the initial period of loneliness [29,30,31,32,33]. After becoming established in the society of the immigrant-receiving place, the immigrant’s ability to learn the language, culture, and customs, find a job in mainstream society, strengthen communication with mainstream social groups, and form a new support network are important factors in the decision as to whether to stay. If immigrants restrict their activities and living circles to their original ethnic group, then they may never integrate into mainstream society and will be excluded from the local society, which may eventually affect their willingness to settle down. If immigrants expand their circle, then they will be able to enter, receive recognition from and integrate into mainstream society, obtain various resources and services from the local society, and, finally, psychologically identify with the local society and gain the local identity.

Many studies have revealed a possible nonlinear relationship between age and willingness to settle down. With increasing age, the settlement intention increases, but after a certain age, the settlement intention decreases [34]. This may be because young people have better social-adaptation abilities and are more willing to accept challenges, while older people’s abilities begin to decline in all aspects. When older individuals are hindered in the immigrant-receiving places, they are more inclined to return to their hometowns. In terms of marital status, there is no agreement regarding its effect on immigrants’ willingness to settle down. Some scholars believe that, when confronted with migration and remigration, married people consider various issues, such as the separation of the family members and children’s education, and so they face more restrictive factors than unmarried people, with a resulting negative impact on the decision of residence [27,35,36,37]. Other scholars reckon that the economic benefits brought about by migration and remigration may far outweigh the losses caused by family separation and do not have a significant impact on migration and residence [38,39,40,41].

From the perspective of the institutional setting, social institutions and policies may be significant variables that affect immigrants’ future settlement intentions. Numerous studies from China on the willingness of the floating population to settle down in cities have revealed how China’s household registration system and barriers reduce the willingness of the floating population to settle down in cities [6,42,43]. Studies on international migration also reveal how the rigidity of the immigration system and policies in the society of the immigrant-receiving place affect immigrants’ integration into society and ultimately influence their willingness to settle down permanently [5].

Although these studies explore many factors that influence the settlement intentions of immigrants from different perspectives, they do not address an interesting phenomenon that widely exists both in the domestic floating population and among international migrants, which is that the health status of migrants who settle down in immigrant-receiving places is better than that of those who move back, and even better than that of local residents. For example, Hispanic immigrants in the United States are healthier than native Americans, they are less likely to suffer from chronic diseases and mental disorders, and they have a lower mortality rate [43,44,45,46]. In contrast, the health status of those who have returned to their hometowns is relatively worse, which cannot be effectively explained from the above research perspectives.

### 1.2. Health, Migration, and Long-Term Settlement Intention

Scholars have proposed two hypotheses to explain this paradox. The first hypothesis is the “healthy migrant” hypothesis, which states that migrants represent a positively selected group of individuals with respect to health, relative to the general populations in origin societies [27,47]. This selection process makes immigrants stand out in terms of health levels when compared with the general populations in destination countries [21]. This implies that healthy immigrants are more willing to migrate than less healthy immigrants [27,43,48]. This hypothesis highlights the important role of health in migration decisions. Why is health so significant for migration? This is because the process of migration is often difficult and consumes physical strength, and it interrupts individuals’ normal lives and requires them to readapt to the society to which they move. The process of migration is painful, and those in poor health are less willing to migrate. In addition, immigrants are often required to undertake manual labor in the immigrant-receiving society. Those who are healthier, more tolerant of such jobs, and financially successful are more willing to migrate. In theory, international migration poses greater barriers to immigrants, and it disrupts social networks more seriously than domestic migration, which may lead to the tendency of international immigrants to have better health. Of the large number of empirical studies that have tested this hypothesis, some have confirmed it [14,49], and others have only partially confirmed it [50].

The second hypothesis is the “salmon bias” effect, or selective return migration, which postulates that immigrants experiencing deteriorating health have a greater tendency to return or move to the place of emigration than healthier migrants [14,49,51]. This hypothesis notes the important influence of health on immigrants’ willingness to settle down. Immigrants who face health problems are limited in their ability to perform efficient work in the local society, which results in a decline in income and living standards. These factors, together with other restrictive factors, such as the lack of social security facilities and social support networks, encourage them to decide to return to their hometowns, or places close to their hometowns, rather than remain in the local society. Ullmann et al. found that immigrants returning to Mexico from the United States had a higher prevalence of chronic diseases than nonimmigrants, such as obesity, lung disease, or heart disease, but there was no difference in the prevalence of other diseases, such as hypertension and diabetes [52].

Many empirical studies have been conducted to test these two hypotheses. For example, Turra and Elo proposed a method to directly evaluate the salmon bias of immigrants from Spain to the United States, and only limited evidence supported this hypothesis [14]. In contrast, Sander studied immigrants returning to Turkey from Germany and found that men in poor health were less likely to return than men in good health, which was considered evidence of the decline in the health status of immigrants in the destination [53]. In China, there are many studies on the domestic floating population that directly or indirectly test the above two hypotheses. For instance, Chen found the existence of the healthy-migrant effect by comparing the floating population and local residents in Beijing [54]. Tong and Piotrowski found that healthy migrants were more likely to stay in the city, although the relationship between health and migration diminishes across time [55]. Lu and Qin compared the self-rated health of the returning and the settled floating populations and found that the results supported both the “health migrant” hypothesis and the salmon-bias effect [21].

Through the analysis of China’s urban–rural mobility data, Xie et al. found that self-evaluated physical and mental health is an important factor that influences the permanent settlement of the floating populations in cities. Moreover, the willingness of the floating population to settle permanently in cities is regulated by the length of their residence [22]. Early studies also revealed that urban and rural migrants have a higher risk of psychological problems. Migrants with psychological disturbance are more willing to return to their hometowns than to settle down in their immigration destination because the social ties in their hometowns can help to ease their mental tension [13,56].

### 1.3. Health Change, Length of Stay, Insurance Status, and Long-Term Settlement Intention

The studies mentioned above all highlight the close relationship between health and immigrants’ settlement intentions. They mostly looked at the relationship between health and settlement from the results, instead of looking at the relationship between changes in health and settlement from a dynamic process.

From the process point of view, health is not changeless but is constantly changing. Many early immigrants worked in the second labor market, with low wages and poor welfare [57,58]; however, this did not mean that immigrants could only work in the low-end labor market. In fact, many highly skilled immigrants were still able to obtain high incomes and high welfare in the immigrant-receiving country [8,59]. Immigrants who arrive at their destinations in poor health at the beginning may not necessarily need to work in the high-intensity manual sector but may engage in mental work in the sectors with relatively low physical demands. They may work well and obtain better income returns and better medical insurance, and thus acquire mainstream social security resources. The good economic conditions and social security level may make them healthier and more willing to settle down in their destination.

In contrast, people who are initially healthy may suffer from physical and mental exhaustion caused by high-intensity labor and constant social competition, which thus results in the deterioration of health [60]. Moreover, if immigrants suffer repulsion and discrimination in the local society, or even systemic exclusion, the process of integration may not be successful, which will also bring about psychological pressure. This may further cause serious psychological problems [61,62]. If the costs of poor health outweigh the benefits of migration behavior, then immigrants may return to their hometowns. Thus, changes in health status rather than health itself may affect settlement intentions. Therefore, we propose the following:

**Hypothesis** **1.** *The change in health status rather than health itself affects an immigrant’s willingness to settle down. The healthier people become, the more likely they are to settle down, and the poorer health they develop, the more likely they are to leave*.

Social insurance is often closely related to people’s health and health changes. People with insurance are more likely to pay for medical expenses and maintain good health conditions. In addition, people with insurance are more likely to have better health status because of their access to relatively better medical services [20,63,64,65,66,67,68]. Anyone can become sick in immigrant-receiving places, and some people may develop chronic diseases, such as hypertension. These all require medical treatment. However, with the improvement in medical services and the progress of medical technology, possibly, the cost of medical services may increase. If there is no local social security or social insurance, people have to pay all the medical fees in full, which is usually expensive. These costs occupy normal living expenses and bring about a heavy burden. In addition, the treatment of diseases involves different treatment plans and means according to an individual’s economic constraints. The lower the economic constraints are, the better the medical services generally are. Therefore, the existence of medical insurance in the immigrant-receiving place can not only relieve the burden posed by medical care, but also enable immigrants to obtain better medical resources in the immigrant-receiving place and thus maintain their standard of living. In contrast, if there is no such social security and insurance, when immigrants need medical services, they will be unable to accept the necessary medical services due to economic constraints, which will lead to deterioration in their health. They will eventually be abandoned by the labor market in the local society, and they will then be unable to survive and will have to leave. Additionally, medical treatment occupies limited living resources and leads to the decline in living standards in the place of migration. This contradicts the initial goal of obtaining a good life through migration and facilitates the decision to leave [69]. Furthermore, the ultimate health levels of people with medical insurance are sure to be higher than those of people who do not have insurance because they can obtain relatively good medical services. This means that even people who have poor health initially may become healthier if they have medical insurance because they can receive good medical services. People who are in good health initially can receive better treatment whenever they become sick and can thus maintain their health status, which supports the decision to continue to settle down. In contrast, people who are initially in poor health receive limited medical services if they lack medical insurance, which can lead to a deterioration in health status. People who are in good health initially may develop health problems due to the pressure of high-intensity labor and social competition, and their health status may then deteriorate due to the lack of effective and timely medical treatment. Finally, they cannot continue to work and live in the local society and must choose to leave. As a result, whether or not immigrants have medical insurance can eventually widen the gap in the decision to stay or leave between those whose health improves and those whose health declines. Thus, we propose the following:

**Hypothesis** **2.** *The presence or absence of medical insurance moderates the impact of changes in health status on the willingness to settle*.

The length of stay can reflect the social integration status of immigrants to some extent. People who are not well integrated may leave the local society early, while those who are well integrated may work, reside, and live in the local society for a long time. In addition, those who live in the local society for a long time not only enjoy good living conditions, including good material conditions and a good living environment, but they are also highly dependent on the medical services of the local society. The former provides the physical foundation for health, and the latter helps cope with the challenges of various diseases. As a result, immigrants who live in their destination for a long time are usually able to maintain or even improve their health, and they are thus better able to adapt to the local society, which, in turn, promotes their willingness to settle down. Although people who stayed longer may have settled, this still cannot hide the fact that, compared with people who stayed for a short time, they are better at using the existing conditions to promote health and are further more likely to settle. Related research has explored that the length of stay will moderate the effect of health on the settlement intention [22]. Following this logic, we believe that the moderation effects of health changes on settlement intentions should also exist.

Therefore, we propose the following:

**Hypothesis** **3.** *Length of stay moderates the impact of changes in health status on settlement intention*.

## 2. Method

### 2.1. Yiwu as a New Host City for International Migrants

Yiwu is a county-level city in the middle of Zhejiang Province. It has a historical tradition of conducting business, but there is no tradition of foreign trade. After the reform and opening up, due to the development of the market economy, it became a global commodity trading center in just a few decades. Although Yiwu is only a small and medium-sized city, nearly 500,000 overseas merchants come to Yiwu to purchase goods every year, and more than 13,000 overseas merchants live in Yiwu [70]. Currently, the foreigners registered in Yiwu come from nearly 219 countries and regions around the world, and mainly from the Middle East, Africa, India in South Asia, South Korea, Japan, and South America in East Asia. There are also merchants from Europe, Latin America, North America, and Oceania. These immigrants come not only from developed countries, but also from less developed countries. Some of them have received higher education, while others may have received limited education. In addition, some of them have high incomes, while others’ incomes are relatively low. The sources of foreigners are extensive. Therefore, Yiwu is a good place for research on international migrants. Not only does it provide the diversity of sources of foreigners, but also, due to their socioeconomic status, varies by their identity.

### 2.2. Data Collection

The data used in this study come from the Yiwu subsample of the Survey of Foreigners Residing in China (SFRC). The project, established in 2016 by the Institute of State Governance of Sun Yat-sen University, expanded from the initial Guangzhou survey in 2016–2017 to include six more cities in 2018 and 2019: Hangzhou, Xi’an, Changchun, Lanzhou, Yiwu, and Xuzhou [19]. In particular, the data used in this study were collected by the authors’ research group at Zhejiang Normal University over approximately four weeks in July and August of 2018 and 2019.

The survey was conducted via a questionnaire administered onsite at the primary exit–entry administration offices of different cities. Participants were recruited through a convenience sampling method, and specifically in the reception halls of the exit–entry administration bureaus. Each participant recruited was briefly introduced to the aims of and the confidentiality policy on the data collected before he/she was invited to voluntarily participate. Data on the participants’ sociodemographic characteristics, job status and employment, immigration experience, social life, and health situation were collected [71].

Since its expansion in 2018, the project has conducted two additional waves of surveys in more cities (in 2018 and 2019). A total of 1206 questionnaires in 2018 and 998 questionnaires in 2019 were collected in Yiwu. After careful examination, 1108 valid samples and 980 valid samples, were obtained separately. Due to the high similarity of the questionnaires used in these two waves of surveys, we combined them to obtain a larger sample of 2088 cases.

### 2.3. Measures

#### 2.3.1. Dependent Variable

The dependent variable used in this paper was measured by the question: “How long you would like to stay in China?” The possible answers were to leave as soon as possible, stay as long as possible, permanently reside in China, and others. In the analysis, we coded permanently reside and stay as long as possible as 1 and leave as soon as possible as 0; the others were deleted in the analysis due to the fact that very few participants chose them.

#### 2.3.2. Explanatory Variables

Changes in health status. To understand the change in the immigrants’ health status, we asked them to indicate the change in their health status during their residence in China. Five different answers were possible: “become very unhealthy”, “become unhealthier than before”, “the same as before”, “become healthier than before”, and “become very healthy”. We coded them from 1 to 5.

Medical insurance. Regarding the medical insurance situation, the immigrants were asked to answer whether they had any medical insurance during their stay in China. The possible answers were “no”, “yes”, and “do not know”. Due to the limited number of participants who answered “do not know”, we directly deleted the cases with this answer in the model. Then, we changed this variable into a dichotomous variable and coded “yes” as 1 and “no” as 0.

Employment status. Respondents were asked whether they had a job in China. “Yes” and “no” were provided as the answers, and, in the analysis, “yes” was coded as 1, and “no” was coded as 0. The variable was treated as a dichotomous variable.

Length of stay in China. This variable was measured by the question on how many months the participants had resided in China. No answers were provided, and the respondents had to fill in a number by themselves. In the analysis, we took 6 months as a unit and recoded no more than 6 months as 1; from 7 to 12 months as 2; from 13 to 18 months as 3; from 17 to 24 months as 4; from 25 to 36 months as 5; 37 months and more as 6.

#### 2.3.3. Control Variables

Health condition. In order to explore the influence of changes in health on settlement intentions, we needed to control for the respondents’ health status at the time of the investigation. The respondents were asked whether they had any existing health problems at that time. The answers were yes and no, which makes this a dichotomous variable. We coded “yes” as 1 and “no” as 0.

In addition, there were other control variables. Gender was handled as a dichotomous variable (male: 0, female: 1). Educational level was coded from 1 to 5, corresponding to primary school or below, junior high school, senior high school, college, and bachelor’s degree or higher. Age is a grouping variable and was divided into: under 20 years old; 21–30 years old; 31–40 years old; 41–50 years old; over 50 years old. These were handled as nominal variables. For income, the respondents were categorized into 5 groups: below CNY 2000, CNY 2001–3000, CNY 3001–5000, CNY 5001–8000, and above CNY 8000. Regional variables were categorized into Middle East, Africa, Europe and America, and Asia, according to the geographical location of the immigrant’s country of origin.

### 2.4. Data Analysis Strategy

This paper first uses the descriptive statistical method to analyze immigrants’ settlement intentions, and then the binary logistic regression method [72] to establish a multivariate analysis model of immigrants’ settlement intentions. The functional relationship between the explained variables and explanatory variables in this model is as follows:

$$\ln\frac{p}{1-p}=\alpha +\beta_{1}x_{1}+\beta_{2}x_{2}+\ldots \ldots +\beta_{k}X_{k}$$

where p is the probability of long-term residence intention, X is the explanatory variable, β is the estimated coefficient of each explanatory variable, and α is a constant term. The value of OR is exp (β), which is equal to p/1 − p. It measures the ratio of the probability of “permanent residence” to “not permanent residence”. Exp (β) can directly compare the differences between groups of explanatory variables and the level of influence of the explanatory variables on the explained variables.

At the same time, in order to show more vividly the marginal moderation effects of the length of stay and medical insurance on the influence of health changes on the settlement intention, we used STATA16.0 to draw the marginal moderation effect.

## 3. Results

### 3.1. Descriptive Analysis Results

According to the data (Table 1), 1375 people, which is approximately 68.31% of the immigrants surveyed, wanted to stay in China permanently, and 638 people, approximately 31.7%, had decided to leave Yiwu. This means that most of the immigrants currently living in Yiwu chose to stay, while only a few people chose to leave.

In terms of gender, the majority of the sample was male; there were 1681 men, accounting for approximately 89.6%, and 215 women, accounting for 10.4% (The imbalance between men and women in Yiwu is not a strange phenomenon. We guess that there are two possible reasons. First of all, according to the law of immigration, in the early stages of immigration, men usually emigrate first, and then take their wives and children to the place of emigration. Yiwu is in the early stage of immigration, and men have an absolute advantage. In addition, the vast majority of immigrants in Yiwu are Muslims. Unmarried Muslim women usually do not start their own businesses. Married women usually migrate with their husbands, but husbands generally consider bringing their wives in after their work and lives are stable, and this takes time. Thus, it is no surprise that the number of male foreigners is several times that of women currently in the initial stage of immigration to Yiwu compared with other places, and especially those places that have long histories of immigration). In terms of age, 3.5% of the people were under the age of 20 years, 42.6% of the people were 21–30 years old, 34.9% of the people were 31–40 years old, 13.4% of the people were 41–50 years old (13.4%), and 5.6% of the people were over 50 years old (5.6%). In sum, young adults made up the majority of immigrants coming to Yiwu, while the oldest and youngest were relatively few. In terms of educational level, 34.9% of the people had received senior high school education, 13.4% of the people had received college, and 5.6% of the people were undergraduates and postgraduates. This showed that, among the immigrants coming to China, most had a middle or high school educational level. In terms of monthly income, more than half of the immigrants earned over RMB 5000, and, among them, 22.56% earned RMB 5001–8000, and 33.27% earned above RMB 8000.

In terms of the employment status of the immigrants surveyed in China, the vast majority had jobs (68.1%). Approximately 20.4% of those surveyed had health problems. Approximately 42.4% had social insurance, approximately 44.3% were uninsured, and approximately 13.1% were not sure whether they had insurance or not. Regarding the change in health status since their immigration to China, approximately 9.3% of people had become very healthy, approximately 23.3% had gained better health, 58.3% had not experienced a change in health, 6.9% had suffered worse health, and 2.2% had become very unhealthy. In terms of the residence time, the average residence time of the immigrants was approximately 19.8 months. If we take 6 months as a unit, approximately 48.6% of people had resided for 6 months, 17.8% for 12 months, 4.3% for 18 months, 8.4% for 24 months, 1.9% for 30 months, 5.6% for 36 months, and 13.4% for more than 36 months.

### 3.2. Logistic Regression Results of Settlement Intention

We built basic Model 1 to explore the relationship between the demographic variables and settlement intentions. Then, in order to demonstrate the relationships between health change, insurance, and length of stay in China, we built another four models. Furthermore, to estimate the moderating role of insurance and length of stay on the effect of health change on the settlement intention, we built three more models.

From the results of Model 1 (Table 2), there was no significant difference between men and women in settlement intentions, but there was a significant difference by age group. People over 30 years old were more willing to leave China, while people under 30 years were more willing to stay in China. There are no significant differences in settlement intentions by income group or educational level. Among the socioeconomic variables, whether an individual had a job affected their willingness to stay in China. People without jobs are more likely to leave China, while those who have jobs are more likely to stay. Compared with the reference group, Africans, Asians, and Middle Easterners were more willing to settle down.

Model 2 tested whether health problems affect the settlement intention. The result showed that whether having health problems or not does not influence the settlement intention. The results of Model 3 revealed that health changes significantly affected the participants’ settlement intentions: those people whose health had become better in one unit were 26% (e^0.233^−1) more likely to settle down compared with those whose health had become worse.

The results of Model 4 demonstrated that the status of medical insurance significantly affected the participants’ settlement intentions. If they had medical insurance in China, then they were more likely to settle down. Specifically, those with health insurance were 1.57 (e^0.452^) times more likely to stay than those without health insurance. While the result of Model 5 showed that there was a close relationship between the length of stay in China and their settlement intentions, for those who had stayed in China, for every additional half-year stay, the probability of the willingness to settle down increased by 10% (e^0.0975^−1).

By combining the above analysis results, we conclude that it is not whether individuals have health problems or not, but rather the change in their health status that is the important factor that affects the settlement intention. If their health status improves, then individuals are more inclined to stay in China, and if their health status deteriorates, then they are more likely to leave China. Moreover, those with medical insurance choose to stay in China, while those without medical insurance choose to leave. This shows that Hypothesis 1 is confirmed.

The results of the above model were analyzed for all samplings including Asians, Middle Easterners, and Africans. However, it is not known whether these results are applicable to different subgroups, or whether there are differences between them. In addition, the overall model shows that there are significant differences in the willingness to settle down between Asians, Middle Easterners, and Africans, which guided us to make a more detailed analysis. In order to test the applicability of these results in different subgroups and explore the possible differences between them, we constructed submodels for Asians, Middle Easterners, and Africans separately (Table 3; details can be found in the Supplementary Files).

According to the submodels by geographical origin, the results for the immigrants from Africa, the Middle East, and Asia are basically the same as those for the overall sample, except for some differences in a few variables (the number of participants from Europe and America was too small, and so we did not establish a model for this group). For example, in the model including the survey respondents from Africa and the Middle East (Model 6a), there is no difference in the willingness to settle down, regardless of age, which differs from the result for the full model. The Asia submodel reveals that only those 21–30 years old are more inclined to settle down, compared with the reference group. Moreover, for the Asia group, those who do not have jobs are less likely to settle down compared with those who have jobs in China, while, for the Africa and Middle East groups, there is no difference between them.

In conclusion, the impacts of the core explanatory variables, such as health change, insurance status, length of stay in China, etc., on the settlement intention are completely consistent with the full model, but there is a slight difference in the impacts of the control variables.

### 3.3. The Effects of Moderating Variables: Medical Insurance and Length of Stay in China

According to the above reasoning, the length of stay and the availability of medical insurance are likely to moderate the influence of health changes on settlement intentions. For this reason, we tested Hypotheses 2 and 3 to confirm whether the results were in accordance with the full model, and, in addition, to explore the possible differences among the different submodels. We constructed an overall model and submodels with the length of stay and medical insurance as the moderating variables (Table 4).

**Figure 1:**
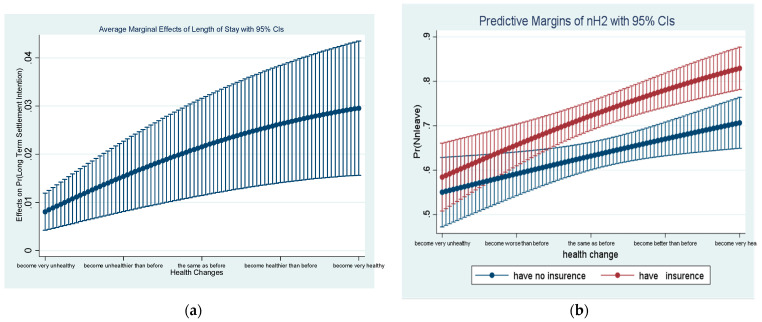
The moderating effects of length of stay in China and insurance status on the association between health change and long-term settlement intention in China: (**a**) moderating effect of length of stay; (**b**) moderating effect of insurance status.

For the overall Model 2, which includes having insurance as the moderating variable, the results show that the change in health status is moderated by medical insurance, and this further affects people’s willingness to settle down. Specifically, for each unit of improvement in health status, there is a difference of approximately 36.1% (e^0.174+0.134^−1) log odds in the settlement intentions between the insured and the uninsured. Moreover, changes in health status are also moderated by the length of stay in China to affect the settlement intention. With one unit increase in the length of stay, the log odds of the influence of changes in health status on the settlement intention will be increased by 19.6% (e^0.149+0.0320^−1). The margin moderating effect of the length of stay and insurance status on the association between changes in health status and long-term settlement intentions is shown in Figure 1. These results reveal that Hypotheses 2 and 3 are confirmed.

The results for the submodels including people from Africa and the Middle East (2a and 3a) are fully consistent with those of the overall model. The major factor that affects the settlement intention is the change in health status, and it is moderated by medical insurance and the length of stay in China. Specifically, for Africans, for every unit of improvement in health status, those who are insured are 109% (e^0.271+0.461^−1) more likely to settle down than those who are uninsured. For each additional unit of staying time in China, the log odds of the influence of changes in health status on settlement in China increased by 62.3% (e^0.427+0.0574^−1). For Middle Easterners, insurance moderates the positive effect of health change on willingness to stay. At the same time, the length of stay also moderates the positive effect of health change on willingness to stay in China, while the Asia model is partially consistent with the full model. Health change is only moderated by medical insurance and is not moderated by the length of stay to affect the settlement intention.

In sum, the results of the Africa submodels are completely consistent with the results of the overall model. As for the Middle Easterners, insurance and length of stay moderate the effect of health change on willingness to stay, while, for the Asians, medical insurance rather than length of stay moderates the effect of health change on the willingness to stay. By combining the results of the overall model and submodels, we reckon that health-status change is the main influencing variable that affects the settlement intention, and the availability of medical insurance and the length of stay probably moderate the influence of health status on settlement intentions according to different areas.

## 4. Discussion

In the past, research on the resettlement intentions of immigrants focused mainly on socioeconomic factors or cultural factors [5,6,15,25,26,27,34,38]. The findings of this study are consistent with those of previous studies, which reconfirms the impact of economic factors, such as having a job, on long-term settlement intentions. Only a small number of studies have focused on the relationship between health and settlement intentions [14,21,22,55]. The healthy-migrant hypothesis and the salmon-bias hypothesis are the most representative theories provided to explain the relationship between health and the willingness to settle. The commonality of these two hypotheses is to explain the relationship between health and the willingness to settle from the perspective of the results, but it is more meaningful to explain the relationship between them from the perspective of the dynamic process of change.

This study uses empirical survey data from China to reveal the relationship between changes in health status and willingness to settle down from the perspective of the process of dynamic changes. The results show that health status alone does not affect immigrants’ willingness to settle in China, but changes in health status have a significant influence on it. This result is slightly inconsistent with the conclusion of previous studies that the health of immigrants (including physical health and mental health) significantly affects their settlement intentions, and that the healthier they are physically and mentally, the more likely they are to stay [22]. The previous studies reveal that if immigrants are not healthy enough, then they cannot be qualified for high-intensity labor in the local society, and so they will eventually be driven out and will leave the local society. If they are not in good mental condition, then they can obtain more social support and help when they return to their hometowns, or places close to their hometowns. However, we believe that there are two defects in the conclusions of previous studies. First, the data used in almost all of the studies are from cross-sectional surveys rather than longitudinal surveys, and many key variables, such as health status, were measured only at the time of the survey. Health status is likely to be a consequence of immigration rather than a cause of immigration. Therefore, the variables themselves have an observational selectivity; that is, almost all of the immigrants who finally stay and settle down are healthy. Second, previous studies tend to explore the relationship between health and the settlement intention from the perspective of results, and little attention is paid to the relationship between changes in health status and the settlement intention from a process perspective.

Our study probably improves the research on this topic in at least two aspects. First, this study posits that health status is not unchanging but is, rather, constantly changing. Those who arrive at their destinations with poor health do not need to work in sectors with high physical intensity but can engage in mental labor in sectors requiring relatively little physical effort, and they may gain sufficient income returns and medical insurance to obtain social security resources in mainstream society. In contrast, immigrants who are initially healthy may become poor after working and living in the destination for a long time. If they cannot obtain social security and medical services due to challenges in the system or their own conditions, they may return to their hometowns. The analysis of the survey data confirms our hypothesis. It shows that it is not the health status itself but the change in health status that affects the willingness to settle down. Second, our core variable is the change in health status, which enables the measurement of the differences between health conditions. Even if the observation-bias effect cannot be completely eliminated, this variable can objectively reflect the changes over time to a certain extent, which is an alternative method when longitudinal data are not available.

Previous studies found that the length of stay moderated the effect of the health status on the intention to settle [22], and the present study follows this line of thought to explore the relationships between the length of stay of immigrants in China, medical insurance status, status of health change, and settlement intention. It reveals that the length of stay of immigrants in China and medical insurance status moderate the impact of health changes on the willingness to settle. We believe that there is a dynamic behind this. Changes in physical health do not usually occur within a short period of time but, rather, represent a process of slow change. In this long-term process, medical insurance status determines whether those who are not in good health initially can have the opportunity to obtain high-quality medical resources, thereby making their health conditions better.

In addition, even people with good physical health face different levels of health problems as they age under work and life pressures [13,54,73]. Whether they have medical insurance determines whether they have the opportunity to receive excellent medical resources and timely assistance in order to maintain or improve their health; if they have no medical insurance, their health condition is likely to grow worse, which thereby affects their work and life. Ultimately, they may be unable to adapt to the local society and may have to leave. Therefore, whether immigrants have medical insurance moderates the impact of the changes in health status on their willingness to settle.

The length of stay in China can be regarded as an important indicator of social adaptation. Generally, the better-adapted immigrants are, the longer they may stay in China, while the less adapted they are, the more likely they are to make an early departure. Regardless of the economic or cultural level of well-adjusted persons, if their own abilities are relatively strong, not only can they maintain their health and make it better, but they may also have a strong ability to use social resources to solve problems. Therefore, the longer immigrants stay, the more likely it is that their health status will be maintained or become even better. The more immigrants adapt to society, the more willing they are to stay. In contrast, if immigrants cannot adapt to the local society, the more likely they are to leave.

Simply, this research contributes to the existing research on immigrants’ settlement intentions. It reveals the impact of health changes on the willingness to settle, which is seldom mentioned, and it also shows how the influence of health changes on settlement intentions is moderated by the length of stay and insurance status.

However, due to the limitation of the data, this study did not distinguish changes in physical health status from changes in mental health status in the analysis. This makes it impossible for us to more precisely distinguish whether and how changes in physical and mental health status differently affect immigrants’ willingness to settle down. Subsequent studies can further differentiate these factors to clarify whether and how their influence differs. In addition, this survey focused on international immigrants. Due to the imperfection of the immigration laws and administration systems, various data about international immigrants had not been disclosed to the public, and so we could not make a sampling frame, or analyze foreign immigrants through strict random sampling, and only the convenience sampling method could be used for sampling. Moreover, it is possible that the overall education and income levels of the samples used for analysis were relatively high, and that the proportion of males was much larger than that of females, which might lead to over-representation. Future research should obtain more information about immigrants, and then use more scientific methods to draw samples.

The above research conclusions also have significant policy implications for the introduction of global high-end talent in the future. At present, with the advancement of globalization, the competition for talent in various countries is very fierce. Traditional Western developed countries, such as the United Kingdom, the United States, and France, formulated policies early to attract talented individuals. Currently, China is also realizing the significance of global talent introduction, and it is committed to introducing talent through various policies and laws. However, health factors may not be currently considered with regard to the attraction of immigrants. This study shows that the change in health status is an important factor that affects immigrants’ decisions as to whether to stay or leave and, likewise, social security status significantly affects their willingness to settle down. Hence, we believe that factors such as health should be taken into consideration in future policies that are designed to attract talent. There are at least two ways to go about this. First, the scope of medical insurance should be expanded to include all immigrants, whether they have permanent residency or not. The promotion of insurance will not only contribute to solving the problems associated with seeing a doctor but will also lay a foundation for their long-term health. Second, society must be committed to improving the environment while advancing economic and social development. The improvement in the environment will reduce the incidences of various diseases, which is conducive to the health of all people. In fact, when high-end talents are looking for a place to move, one of the most vital aspects that they consider is the natural environment, and places with low levels of pollution tend to receive their attention. Finally, a psychological consulting industry should be developed. The fast-paced and fierce social competition can easily cause many psychological problems. Thus, it is necessary to carry out relevant psychological interventions to not only relieve the pressure that immigrants face, but also support their mental health.

## 5. Conclusions

This study examined the relationships between the status of health change, length of stay, medical status, and international migrants’ long-term settlement intentions. The results indicate that the status of health change, rather than health status, significantly influenced international migrants’ long-term settlement intentions, and the length of stay and medical insurance status moderated the effect of health changes on settlement intentions in general. However, there are differences between different groups. For Middle Easterners, insurance and length of stay moderate the effect of health changes on the willingness to stay, while, for Asians, medical insurance rather than length of stay moderates the effect of health changes on the willingness to stay.

## Figures and Tables

**Table 1:** Description of variables.

Variables	Frequency	Percentage	Variables	Frequency	Percentage
Long-term settlement intention			Health problem		
Yes	1375	68.3%	Yes	418	20.4%
No	638	31.7%	No	1634	79.6%
Gender			Social insurance		
Male	1681	89.6%	Yes	976	42.5%
Female	215	10.4%	No	915	44.4%
Age group			Have no idea	271	13.1%
Under 20 years old	69	3.5%	Health change		
21–30 years old	841	42.6%	Become very unhealthy	45	2.2%
31–40 years old	689	34.9%	Become worse than before	143	6.9%
41–50 years old	264	13.3%	The same as before	1204	58.3%
Over 50 years old	111	5.6%	Become better than before	482	23.3%
Education			Become very healthy	191	9.3%
Primary school	69	3.5%	Length of stay in China		
Junior high school	841	42.6%	Less than 6 months	904	48.6%
Senior high school	689	34.9%	From 6 to12 months	331	17.8%
College	264	13.4%	From 13 to 18 months	80	4.3%
Undergraduate and above	111	5.6%	From 19 to 24 months	156	8.4%
Income			From 25 to 32 months	35	1.9%
Below CNY 2000	128	6.4%	From 33 to 36 months	105	5.6%
CNY 2001–3000	396	19.6%	37 months and above	250	13.4%
CNY 3001–5000	367	18.2%	Employment status		
CNY 5001–8000	455	22.6%	Yes	1405	68.1%
Above CNY 8000	671	33.3%	No	658	31.9%

**Table 2:** Regression analysis of long-term settlement intention.

VARIABLES	Model 1	Model 2	Model 3	Model 4	Model 5
Sex					
(Male = 0)	0.109	0.113	0.147	0.183	0.166
	(0.189)	(0.190)	(0.191)	(0.192)	(0.209)
Education Level (Primary school = 0)					
Junior high school	−0.0955	−0.125	−0.138	−0.0464	0.0113
	(0.711)	(0.713)	(0.715)	(0.723)	(0.726)
Senior high school	0.188	0.187	0.153	0.234	0.214
	(0.667)	(0.668)	(0.669)	(0.677)	(0.685)
Junior college	−0.0736	−0.0700	−0.0887	−0.0132	0.0474
	(0.619)	(0.621)	(0.621)	(0.628)	(0.633)
Bachelor or above	−0.0706	−0.0789	−0.0867	−0.0469	0.0336
	(0.610)	(0.611)	(0.611)	(0.618)	(0.622)
Age group (Under 20 years old = 0)					
21–30 years old	−0.103	−0.101	−0.0623	−0.0375	−0.0512
	(0.311)	(0.311)	(0.314)	(0.315)	(0.344)
31–40 years old	−0.619 *	−0.609	−0.583	−0.552	−0.614
	(0.315)	(0.315)	(0.317)	(0.318)	(0.347)
41–50 years old	−0.967 **	−0.955 **	−0.930 **	−0.848 *	−0.832 *
	(0.333)	(0.334)	(0.336)	(0.338)	(0.369)
Over 50 years old	−1.114 **	−1.096 **	−1.090 **	−1.036 **	−1.095 **
	(0.363)	(0.364)	(0.366)	(0.368)	(0.400)
Income group (Below CNY 1000 = 0)					
CNY 1001–3000	0.0154	0.0400	0.0213	0.0540	0.000352
	(0.242)	(0.243)	(0.246)	(0.247)	(0.261)
CNY 3001–5000	0.235	0.237	0.225	0.230	0.212
	(0.246)	(0.247)	(0.249)	(0.250)	(0.265)
CNY 5001–8000	0.0726	0.0957	0.105	0.100	0.0805
	(0.238)	(0.240)	(0.241)	(0.243)	(0.257)
Above CNY 8000	−0.0319	−0.0114	0.00776	0.00215	−0.0104
	(0.231)	(0.232)	(0.234)	(0.235)	(0.248)
No jobs	−0.544 ***	−0.520 ***	−0.508 ***	−0.435 ***	−0.349 **
(Have jobs)	(0.122)	(0.123)	(0.123)	(0.125)	(0.132)
Area (Africa = 0)					
Middle East	0.635 ***	0.624 ***	0.597 ***	0.571 ***	0.560 ***
	(0.139)	(0.141)	(0.142)	(0.143)	(0.152)
Asia	0.603 ***	0.595 ***	0.582 ***	0.552 ***	0.530 **
	(0.150)	(0.150)	(0.151)	(0.152)	(0.162)
No health problems		0.0842	0.152	0.126	0.101
(Have health problem = 0)		(0.138)	(0.140)	(0.142)	(0.150)
Health change			0.233 ***	0.248 ***	0.230 **
			(0.0683)	(0.0690)	(0.0728)
Have medical insurance				0.452 ***	0.430 ***
(No insurance = 0)				(0.111)	(0.120)
Length of stay in China					0.0975 ***
					(0.0278)
Constant	1.506 *	1.357	0.487	0.107	−0.133
	(0.752)	(0.776)	(0.818)	(0.830)	(0.862)
N	1760	1741	1737	1735	1584

* *p* < 0.05, ** *p* < 0.01, *** *p* < 0.001.

**Table 3:** Regression analysis of long-term settlement intention (separated models).

	Model 6a	Model 6b	Model 6c
	Africa	Middle East	Asia
No jobs	−0.0415	−0.341	−0.797 **
(Have jobs = 0)	(0.269)	(0.221)	(0.278)
No health problems	−0.328	0.281	−0.220
(Have health problem = 0)	(0.408)	(0.216)	(0.299)
Health change	0.561 ***	0.157 ***	0.117 ***
	(0.0171)	(0.0108)	(0.0149)
Have medical insurance	0.860 **	0.358 *	0.540 *
(No insurance)	(0.277)	(0.156)	(0.237)
Length of stay in China	0.0918 ***	0.03042 *	0.03918 *
	(−0.03366)	(−0.01551)	−0.01993
Control variables ^+^	Yes	Yes	Yes
Constant	−1.984	−0.129	1.087
	(1.454)	(1.061)	(1.581)
N	331	684	438

* *p* < 0.05, ** *p* < 0.01, *** *p* < 0.001; ^+^ Control variables in these models included age, age 2 education level, marital status, income, etc. More details can be seen in the Supplementary Files.

**Table 4:** Moderating effect in the regression analysis of long-term settlement intention.

Variables	Full Sample	Africa	Middle East	Asia	Full Sample	Africa	Middle East	Asia
Area								
Middle East	−0.347 **				−0.340 *			
	(0.132)				(0.133)			
Asia	0.563 ***				0.559 ***			
	(0.151)				(0.152)			
No jobs	0.534 ***	−0.0920	−0.299	−0.855 ***	0.532 **	−0.0648	−0.306	−0.828 ***
(Have jobs = 0)	(0.162)	(0.269)	(0.222)	(0.278)	(0.162)	(0.269)	(0.222)	(0.279)
No health problems	0.106	−0.355	0.295	−0.226	0.103	−0.355	0.292	−0.242
(Have health problems = 0)	(0.150)	(0.411)	(0.217)	(0.297)	(0.150)	(0.413)	(0.216)	(0.298)
Health change	0.174 *	0.461 ***	0.108	0.0442	0.149 *	0.427 **	0.0807	0.0691
	(0.0733)	(0.174)	(0.109)	(0.147)	(0.0745)	(0.173)	(0.111)	(0.152)
Have medical insurance					0.422 ***	0.845 ***	0.322 *	0.550 **
(No insurance = 0)					(0.120)	(0.278)	(0.188)	(0.239)
Have medical insurance × Health change	0.134 ***	0.271 ***	0.113 **	0.139 *				
	(0.0360)	(0.0847)	(0.0554)	(0.0707)				
Length of stay in China	0.0964 ***	0.169 ***	0.0881 **	0.0477				
	(0.0278)	(0.0654)	(0.0420)	(0.0544)				
Length of stay in China × Health change					0.0320 ***	0.0574 ***	0.0282 **	0.0164
					(0.00853)	(0.0205)	(0.0125)	(0.0165)
Control variables ^+^	Yes	Yes	Yes	Yes	Yes	Yes	Yes	Yes
Constant	0.0550	−1.849	−0.148	1.487	0.118	−1.691	−0.0174	1.378
	(0.860)	(1.483)	(1.059)	(1.569)	(0.857)	(1.473)	(1.057)	(1.572)
N		331	684	438	1632	331	684	438

* *p* < 0.05, ** *p* < 0.01, *** *p* < 0.001; ^+^ Control variables in this model included age, age 2 education level, marital status, etc. More details can be seen in the Supplementary Files.

## Data Availability

The data presented in this study are available upon request from the corresponding author.

## Supplementary Materials

The following supporting information can be downloaded at: https://www.mdpi.com/article/10.3390/ijerph19137574/s1, Table S1: Regression analysis of long-term settlement intention (Separated Models); Table S2: Interaction effect in the regression analysis of long-term settlement intention.
