# Supplementary material for: The Effect of Health Change on Long-Term Settlement Intentions of International Immigrants in New Destination Countries: Evidence from Yiwu City in China
Source: Int J Environ Res Public Health. 2022 Jun 21;19(13):7574. doi: 10.3390/ijerph19137574 (PMC9266219; doi:10.3390/ijerph19137574)
Supplement: Supplementary file 1 [file ijerph-19-07574-s001.zip › ijerph-1673228-supplementary.pdf]

Table S1: Regression analysis of long-term settlement intention(Separated Models)

VARIABLES	Model 6a	Model 6b	Model 6c
	**Africa**	**Middle East**	**Asia**
Sex(Male=0)	0.124	0.299	0.818
	(0.408)	(0.446)	(0.470)
Education			
Junior high school	0.194	0.0508	——
	(0.574)	(0.906)	——
Senior high school	-0.113	0.591	0.109
	(0.799)	(0.768)	(1.266)
Junior college	-0.358	0.630	-0.224
	(0.374)	(0.667)	(1.179)
Bachelor or above	——	0.448	-0.190
(Primary school=0)	——	(0.646)	(1.145)
Age group			
21-30 years old	0.596	0.214	1.317*
	(1.035)	(0.421)	(0.532)
31-40 years old	0.449	-0.337	0.402
	(1.041)	(0.423)	(0.526)
41-50 years old	-0.191	-0.416	0.00786
	(1.076)	(0.473)	(0.559)
over 50 years old	-0.637	-0.829	——
(under 20 years old=0)	(1.247)	(0.501)	——
Income			
1,001-3,000 yuan	0.341	-0.217	-0.551
	(0.565)	(0.389)	(0.577)
3,001-5,000 yuan	-0.0413	0.490	-0.0562
	(0.561)	(0.405)	(0.598)
5,001-8,000 yuan	0.157	0.206	-0.203
	(0.544)	(0.388)	(0.579)
above 8,000 yuan	-0.388	0.0135	-0.188
(below 1,000 yuan=0)	(0.562)	(0.363)	(0.556)
No jobs	-0.0415	-0.341	-0.797**
(have jobs=0)	(0.269)	(0.221)	(0.278)
No health problems	-0.328	0.281	-0.220
(have health problem=0)	(0.408)	(0.216)	(0.299)
Health change	0.561***	0.157***	0.117***
	(0.0171)	(0.0108)	(0.0149)
Have medical insurance	0.860**	0.358*	0.540*

(no insurance)	(0.277)	(0.156)	(0.237)
Length of stay in China	0.0918***	0.03042*	0.03918*
	(-0.03366)	(-0.01551)	-0.01993
Constant	-1.984	-0.129	1.087
	(1.454)	(1.061)	(1.581)
N	331	684	438

* p <0.05, **p<0.01, ***p<0.001

Table S2: Moderating effect in the regression analysis of long-term settlement intention

VARIABLES	(1) **Full sample**	(2) **Africa**	(3) **Middle East**	(5) **Asia**	(6) **Full sample**	(7) **Africa**	(8) **Middle East**	(10) **Asia**
Sex	0.151	0.110	0.235	0.801*	0.164	0.140	0.259	0.828*
(Male=0)	(0.209)	(0.405)	(0.447)	(0.467)	(0.209)	(0.405)	(0.446)	(0.470)
Education Level(Primary school=0)								
Junior high school	-0.00681	0.224	0.0828	——	0.00738	0.190	0.0755	——
	(0.726)	(0.567)	(0.908)	——	(0.725)	(0.574)	(0.907)	——
Senior high school	0.194	-0.165	0.576	0.163	0.211	-0.124	0.567	0.138
	(0.685)	(0.807)	(0.771)	(1.263)	(0.685)	(0.806)	(0.771)	(1.265)
Junior college	0.0442	-0.315	0.639	-0.179	0.0463	-0.329	0.619	-0.236
	(0.633)	(0.370)	(0.670)	(1.180)	(0.632)	(0.370)	(0.670)	(1.180)
Bachelor or above	0.0214	——	0.466	-0.157	0.0361	——	0.460	-0.197
	(0.622)	——	(0.649)	(1.148)	(0.621)	——	(0.649)	(1.147)
Age group(under 20 years old=0)								
21-30 years old	-0.0524	0.680	0.200	1.200**	-0.0684	0.561	0.185	1.207**
	(0.344)	(1.070)	(0.421)	(0.522)	(0.344)	(1.058)	(0.421)	(0.523)
31-40 years old	-0.613	0.533	-0.358	0.300	-0.631	0.429	-0.375	0.299
	(0.347)	(1.075)	(0.423)	(0.516)	(0.347)	(1.065)	(0.423)	(0.518)
41-50 years old	-0.832*	-0.0230	-0.392	-0.0413	-0.845*	-0.105	-0.415	-0.0390

	(0.369)	(1.108)	(0.473)	(0.551)	(0.369)	(1.100)	(0.473)	(0.553)
over 50 years old	-1.093**	-0.524	-0.822	——	-1.113**	-0.633	-0.844*	——
	(0.400)	(1.278)	(0.501)	——	(0.400)	(1.266)	(0.501)	——
Income group (below 1,000 yuan=0)								
1,001-3,000 yuan	0.000891	0.398	-0.251	-0.519	-0.00141	0.382	-0.252	-0.524
	(0.260)	(0.565)	(0.389)	(0.572)	(0.260)	(0.565)	(0.389)	(0.575)
3,001-5,000 yuan	0.208	-0.0335	0.450	-0.0156	0.206	-0.0497	0.454	-0.0294
	(0.265)	(0.563)	(0.406)	(0.594)	(0.265)	(0.564)	(0.405)	(0.596)
5,001-8,000 yuan	0.0811	0.165	0.170	-0.186	0.0812	0.162	0.172	-0.204
	(0.257)	(0.546)	(0.388)	(0.576)	(0.257)	(0.546)	(0.388)	(0.578)
above 8,000 yuan	-0.0125	-0.355	-0.0172	-0.167	-0.00674	-0.343	-0.00593	-0.176
	(0.248)	(0.562)	(0.364)	(0.552)	(0.248)	(0.562)	(0.363)	(0.555)
Area								
Middle East	-0.347**				-0.340*			
	(0.132)				(0.133)			
Asia	0.563***				0.559***			
	(0.151)				(0.152)			
No jobs	0.534***	-0.0920	-0.299	-0.855***	0.532**	-0.0648	-0.306	-0.828***
(have jobs=0)	(0.162)	(0.269)	(0.222)	(0.278)	(0.162)	(0.269)	(0.222)	(0.279)
No health problems	0.106	-0.355	0.295	-0.226	0.103	-0.355	0.292	-0.242
(have health problem=0)	(0.150)	(0.411)	(0.217)	(0.297)	(0.150)	(0.413)	(0.216)	(0.298)
Health change	0.174*	0.461***	0.108	0.0442	0.149*	0.427**	0.0807	0.0691
	(0.0733)	(0.174)	(0.109)	(0.147)	(0.0745)	(0.173)	(0.111)	(0.152)
Have medical insurance					0.422***	0.845***	0.322*	0.550**
(no insurance=0)					(0.120)	(0.278)	(0.188)	(0.239)
Have medical insurance× Health change	0.134***	0.271***	0.113**	0.139*				
	(0.0360)	(0.0847)	(0.0554)	(0.0707)				
Length of stay in China	0.0964***	0.169***	0.0881**	0.0477				
	(0.0278)	(0.0654)	(0.0420)	(0.0544)				
Length of stay					0.0320***	0.0574***	0.0282**	0.0164

in China × Health change								
					(0.00853)	(0.0205)	(0.0125)	(0.0165)
Constant	0.0550	-1.849	-0.148	1.487	0.118	-1.691	-0.0174	1.378
	(0.860)	(1.483)	(1.059)	(1.569)	(0.857)	(1.473)	(1.057)	(1.572)
N		331	684	438	1,632	331	684	438

* p <0.05, **p<0.01, ***p<0.001
